# Regulatory and structural properties differentiating the chromosomal and the bacteriophage-associated *Escherichia coli *O157:H7 Cu, Zn Superoxide Dismutases

**DOI:** 10.1186/1471-2180-8-166

**Published:** 2008-10-01

**Authors:** Melania D'Orazio, Raffaella Scotti, Laura Nicolini, Laura Cervoni, Giuseppe Rotilio, Andrea Battistoni, Roberta Gabbianelli

**Affiliations:** 1 Department of Biology, University of Rome, Tor Vergata, Rome, Italy; 2 Biotechnology Service and Animal Welfare, Istituto Superiore di Sanità, Rome, Italy; 3 Department of Biochemical Sciences, A. Rossi Fanelli, University of Rome, La Sapienza, Rome, Italy; 4 IRCCS San Raffaele, La Pisana, Rome, Italy; 5 Consorzio Interuniversitario, Istituto Nazionale Biostrutture e Biosistemi, Rome, Italy

## Abstract

**Background:**

Highly virulent enterohemorrhagic *Escherichia coli *O157:H7 strains possess three *sodC *genes encoding for periplasmic Cu, Zn superoxide dismutases: *sodC*, which is identical to the gene present in non-pathogenic *E. coli *strains, and *sodC*-F1 and *sodC*-F2, two nearly identical genes located within lambdoid prophage sequences. The significance of this apparent *sodC *redundancy in *E. coli *O157:H7 has not yet been investigated.

**Results:**

We report that strains deleted of one or more *sodC *genes are less resistant than the wild type strain to a challenge with hydrogen peroxide, thus confirming their involvement in the bacterial antioxidant apparatus. To understand if the different *sodC *genes have truly overlapping functions, we have carried out a comparison of the functional, structural and regulatory properties of the various *E. coli *O157:H7 SodC enzymes. We have found that the chromosomal and prophagic *sodC *genes are differentially regulated *in vitro*. *sodC *is exclusively expressed in aerobic cultures grown to the stationary phase. In contrast, *sodC*-F1 and *sodC*-F2 are expressed also in the logarithmic phase and in anaerobic cultures. Moreover, the abundance of SodC-F1/SodC-F2 increases with respect to that of SodC in bacteria recovered from infected Caco-2 cells, suggesting higher expression/stability of SodC-F1/SodC-F2 in intracellular environments. This observation correlates with the properties of the proteins. In fact, monomeric SodC and dimeric SodC-F1/SodC-F2 are characterized by sharp differences in catalytic activity, metal affinity, protease resistance and stability.

**Conclusion:**

Our data show that the chromosomal and bacteriophage-associated *E. coli *O157:H7 *sodC *genes have different regulatory properties and encode for proteins with distinct structural/functional features, suggesting that they likely play distinctive roles in bacterial protection from reactive oxygen species. In particular, dimeric SodC-F1 and SodC-F2 possess physico-chemical properties which make these enzymes more suitable than SodC to resist the harsh environmental conditions which are encountered by bacteria within the infected host.

## Background

Enterohemorrhagic *Escherichia coli *(EHEC), including strains of the highly virulent O157:H7 serotype, is responsible for a wide spectrum of diseases ranging from mild diarrhoea to hemorrhagic colitis and the potentially fatal haemolytic uremic syndrome (HUS) [1]. EHEC colonizes the large intestine mucosa, where it causes characteristic attaching and effacing lesions on intestinal epithelia and produces the potent Shiga toxins which are responsible for the major symptoms of hemorrhagic colitis and HUS [2]. The severity of the disease, the lack of effective treatments to decrease the morbidity and mortality associated to infections and the risks of large-scale outbreaks from contaminated food supplies have stimulated intensive research on the pathogenesis and detection of *E. coli *O157:H7 [3,4]. Shiga toxins play a major role in EHEC pathogenesis, but an increasing number of additional virulence factors has been described in recent years. The sequencing of *E. coli *O157:H7 genomes [3,4] has revealed that several potential virulence-associated genes are carried by mobile genetic elements, such as plasmids or prophages, or are localized within pathogenicity islands. In fact, although this organism shares 4.1 Mb of DNA with *E. coli *K12, it has 1.34 Mb of DNA distributed among 177 DNA segments, termed O islands, that are absent in *E. coli *K12 [3].

Genome analysis have also revealed the presence of three *sodC *genes in *E. coli *O157:H7, encoding for periplasmic copper, zinc superoxide dismutases (Cu,ZnSOD) [3,4]. Cu,ZnSOD is an important component of the antioxidant defence of aerobic organisms which catalyzes the dismutation of the highly reactive superoxide radical anion into oxygen and hydrogen peroxide [5]. In Gram-negative bacteria Cu,ZnSODs are localized in the periplasm [6]. As the negatively charged superoxide anion can not easily cross membranes, it has been suggested that the role of this enzyme is to detoxify the periplasmic space from superoxide generated during aerobic growth [7] and/or to protect bacteria from extracellular sources of reactive oxygen species, such as phagocytic cells [6,8]. Although Cu,ZnSOD is present in several non-pathogenic bacteria, thus indicating that this enzyme has functions unrelated to the host-microbe interaction, different studies have established that it contributes to virulence by protecting pathogens from the oxidative burst of host macrophages ([9] and references therein).

One of the three *sodC *copies of *E. coli *O157:H7 is localized on a chromosomal backbone sequence which is shared with *E. coli *K12 and it is homologous to the *sodC *gene from this microorganism. In contrast, the other two *sodC *genes, which encode nearly identical proteins differing for only one amino acid, are embedded within the sequences of two lambdoid prophages (denominated CP-933R and CP-933V in the EDL933 strain) [3,4].

The presence of multiple *sodC *copies within a single bacterial genome is not unique to *E. coli *O157:H7 and a similar condition has been described in *Salmonella enterica*. In fact, many of most virulent *Salmonella *strains possess a bacteriophage-encoded *sodC *copy (*sodCI*) in addition to the chromosomal *sodCII *gene, which is present in all *Salmonella *strains [10]. A number of studies have established that *sodCI *significantly contributes to *Salmonella *virulence, whereas *sodCII *has a modest role in pathogenesis [9,11-13]. Recent studies have shown that differences in gene regulation and in the activity and stability of the two Cu,ZnSODs account for the major contribution of the bacteriophage-encoded *SodCI *enzyme to *Salmonella *virulence [9]. Based on these studies it is tempting to speculate that the prophage-associated *sodC *genes might play a role in O157:H7 virulence.

In this work, we have undertaken an investigation on the regulation of the different *sodC *genes of *E. coli *O157:H7 and on the structural/functional properties of the encoded proteins. Our results point out to differences in gene regulation and in the physico-chemical properties of the enzymes, suggesting that the phage-associated *sodC *genes encode enzyme variants optimized to resist the harsh environmental conditions encountered by bacteria within the infected host.

## Methods

### Reagents

Antibiotics, bovine serum albumin, isopropyl β-D-thiogalactopyranoside (IPTG), pyrogallol, Triton-X114, Triton-X100 and cell culture products were purchased from Sigma-Aldrich. Restriction endonucleases, DNA-modifying enzyme and the High-Fidelity Expand DNA polymerase were obtained from Roche, Euro *Taq *DNA polymerase were obtained from EuroClone (Milan, Italy), *Pfu *DNA polymerase DNA polymerase from Promega. All other chemicals were purchased from BDH and were of the highest grade available. The oligonucleotides were synthesized by Primm (Milan, Italy).

### Bacterial strains and growth conditions

All the bacterial strains used in this work are listed in Table 1. The *E. coli *O157:H7 ED597 strain is a clinical human isolate connected to a HUS case (kindly provided from Dr. S. Morabito, Department of Food Safety and Veterinary Public Health, Istituto Superiore di Sanità, Rome). Unless otherwise specified, bacteria were grown at 37°C in Luria-Bertani (LB) medium (1% bacto tryptone w/v, 0.5% yeast extract w/v, 1% NaCl w/v) or in LB medium solidified with 1.5% (w/v) agar. Anaerobic growth was achieved in GasPack anaerobic jars using LB broth containing 0.2% glucose. When required, the culture media were supplemented with the appropriate antibiotics (ampicillin 100 μg/ml; kanamycin 50 μg/ml; chloramphenicol 30 μg/ml or 15 μg/ml for *E. coli *K12 and *E. coli *O157:H7, respectively).

**Table 1:** Bacterial strains

**Strains**	**Relevant genotype**	**Reference or source**
***E. coli *K-12**		
71/18	F' *lacI*^q ^Δ(lacZ)M15	Messing *et al.*, 1997
	*pro*A+B+/Δ(*lac-proAB*) *thi supE*	
QC771	F^- ^Δ(*lac-argF*)U169 *rps*L179	Carlioz & Touati 1986
QC1110	QC771 *katF*::Tn10 Tet^r^	Battistoni *et al.*, 2000
QC871	HVC45 (F^- ^l*eu*6TbrA *pro lac*Y1 *sup*E44	Carlioz & Touati 1986
	*Hsd*R *rps*L *Ton*A *Thi sodA*25 *sodB *Δ2	
		
***E. coli *O157:H7**		
EDL933	Wild type	Riley L.W.*et al.*,1983
ED597	Wild type	Morabito S. collection
RG101	Δ*sodC*::*cat*	this study
RG102	Δ*sodC*-F1::*cat*	this study
RG103	Δ*sodC*-F2::*cat*	this study
RG104	Δ*sodC*-F1/Δ*sodC*-F2::*cat*	this study
RG105	Δ*sodC*-F1/Δ*sodC*-F2/Δ*sodC*::*cat*	this study
RG-F106	*sodC*::3xFLAG-*kan*	this study
RG-F107	*sodC*::3xFLAG-*cat*	this study
RG-F108	*sodC*-F1::3xFLAG-*kan*	this study
RG-F109	*sodC*-F2::3xFLAG-*kan*	this study
RG-F110	*sodC*::3xFLAG/*sodC*-F1::3xFLAG-*kan*	this study
RG-F111	*sodC*::3xFLAG-*cat*/*sodC*-F2::3xFLAG-*kan*	this study

### Construction of deletion mutants

In this work the two prophagic *sodC *genes will be designed as *sodC*-F1 and *sodC*-F2. Further details about such a nomenclature are reported in the first paragraph of the results section.

All gene replacement experiments were carried out by the lambda-red-mediated recombination procedure as described by Datsenko and Wanner [14]. Briefly, a fragment containing chloramphenicol resistance cassette was amplified from plasmid pKD3 [14] using primers (H5P1 and H6P2 or H7P1 and H8P2) with sequence extensions complementary to the target sequences (Table 2) and electroporated in the *E. coli *ED597 strain carrying pKD46 [14]. Recombinants were selected on chloramphenicol LB plates and confirmed by PCR using the internal chloramphenicol cassette oligonucleotide Clo-int as reverse primer and oligonucleotides 120ATGcoli-F, 240ATG-F1 or 240ATG-F2 as forward specific primer for the screening of *sod*C, *sod*C-F1 or *sod*C-F2 null mutants, respectively. The mutant strains lacking the chromosomal *sodC *or the prophagic *sodC*-F genes, were designated RG101 (Δ*sodC*::*cat*), RG102 (Δ*sodC*-F1::*cat*) and RG103 (Δ*sodC*-F2::*cat*), respectively.

**Table 2:** Oligonucleotides used

**Primer**	**Sequence (5' – 3')**
H5P1 ^a^	GCGGATACGCAGCAGAACAGGAAGTCCCAATGAACCTTGTCTGTAGGCTGGAGCTGCTTCG
H6P2 ^a^	CGCACCACCACCGCCCAGGGGCTCCGGATGGTCATGATGGCATATGAATATCCTCCTTAG
H7P1 ^b^	GGGCAGTCAATTGGTAGCGTCACCATTACTGAAACCGATATGTAGGCTGGAGCTGCTTCG
H8P2 ^b^	CACCGCCACCGCCCAGCGGTTTAGGTTGATCGGACATATTACATATGAATATCCTCCTTAG
3xFLAG-F ^c^	GGGCGGTGGTGGTGCGAGAATGGCCTGCGGCATCATTCAAGACTACAAAGACCATGACGG
3xFLAG-R2 ^c^	ATGTCACGACAAAAACATTAACTCAGAGAGGGAGGATGTGCCGCATATGAATATCCTCCTTAG
3xFLAGcoli-F ^d^	GGGCGGTGGTGGTGCGAGAATGGCCTGCGGCATCATTCAAGACTACAAAGACCATGACGG
3xFLAGcoli-R ^d^	GTGAGCGTGGCGTTCAGCAAAAATCACTGGCGGCCACACTCATATGAATATCCTCCTTAG
240ATG-F1 ^e^	GACCTTCAATCGGCCCTT
240ATG-F2 ^e^	GGCCTTCTATCGGTCCCT
550F1b ^e^	CCCAGTTACCGTTACGCTGCAA
550F2b ^e^	TCCATTTACCACCACGCTCAAG
500STOP1R ^e^	CGGGGCATCAATGGCGTTATG
500STOP2R ^e^	GGAGCATCGATGGCAGCCTG
120ATGcoli-F^e^	GGTTTCGTATCCGTAAAGCG
300STOPcoli-R ^e^	GGGTATAGTGCTGCTGAACT
Clo-Int ^e^	CTGGATATACCACCGTTGAT
Stop Clo-Int ^e^	CACTCATCGCAGTACTGTT
Kan-F ^e^	TGAACAAGATGGATTGCACG
Kan-R ^e^	AAGAACTCGTCAAGAAGGC
O157-1For	CCGGAATTCTAAATGTAAAATCATTGCTGCC
O157-1Rev	ACAAGCTTTTATTGAATGATGCCGCAGG
O157-2For	ATCCATGGCTGCAGAACAGGAAGTCCCAAT
O157-2Rev	CAGAATTCTTATTGAATGATGCCGCAGG
PromEDLFor	TTGAATTCCCGTGTCCGTCAGCGGGG
PRromEDLRev	TTGGATCCGCCATAAAACCCTCATTAATTC

P1, P2 and 3xFLAG homolog sequences are underlined. (a) primers used to create *sodC*-F1/*sodC*-F2 null mutants, (b) primers used to create *sodC *null mutant; (c) primers used to insert 3xFLAG epitope in *sodC*-F1/*sodC*-F2 genes; (d) primers used to insert 3xFLAG epitope in s*odC *gene; (e) primers used for screening of mutant clones or in southern experiments.

The same procedure was employed to obtain the double Δ*sodC*-F1/Δ*sodC*-F2 prophagic null mutant, after deletion of the chloramphenicol resistance cassette from the strain RG102, using the FLP recombinase encoded by plasmid pCP20 [14]. After electroporation of the same fragment previously used to construct the single Δ*sodC*-F2::*cat *mutant into strain RG102 carrying pKD46, recombinants were selected on chloramphenicol LB plates. Deletion of *sodC*-F2 was confirmed by PCR, using the oligonucleotides Clo-int and 240ATG-F2. The Δ*sodC*-F1/Δ*sodC*-F2 mutant strain was designated RG104. Subsequently, the PCR fragment amplified with H7P1 and H8P2 primers, was electroporated into the RG104 deleted of the antibiotic resistance cassette, in order to construct a triple *sodC *mutant strain. Recombinants were selected on chloramphenicol LB plates and confirmed by PCR using oligonucleotides 120ATGcoli-F and Clo-int. The resulting Δ*sodC*-F1/Δ*so*d*C*-F2/Δ*sodC*::*cat *strain was designated RG105.

To further verify the deletion of the genes, all the knockout strains were analyzed by Southern-blot, in comparison with wild type *E. coli *O157:H7 ED597 (data not shown). Genomic DNAs were digested with *Cla*I, fractioned on 1% agarose gels and blotted onto nitrocellulose membrane (Hybond N, Amersham). PCR fragments obtained with oligonucleotides specific for *sodC*-F1 or *sodC*-F2, *sodC*, and chloramphenicol genes (Table 2) were labelled using digoxigenin DNA labelling and detection kit (Roche). Hybridisation was performed at 49°C. The hybridisation patterns observed in all strains confirmed the correct introduction of mutations.

### Construction of *sodC*-*lacZ *fusions and β-galactosidase assay

To analyze the transcriptional regulation of *sodC *and *sodC-*F genes, the promoter region of *sodC-*F gene (corresponding to the 299 bp before the translation start site) was amplified by PCR using the oligonucleotides PromEDLFor and PromEDLRev (Table 2). The amplified DNA fragment was digested with *Eco*RI and *Bam*HI and inserted upstream of a promoter-less *lacZ *gene into the promoter probe plasmid pMC1403 [15], obtaining pMCPromO157. The construction of plasmid pMCPromEcSOD, obtained by introducing the *E. coli *K12 *sodC *promoter (identical to chromosomal *sodC *promoter by *E. coli *O157:H7) in pMC1403, has been previously described [16].

pMCPromEcSOD and pMCPromO157 were introduced into *E. coli *QC771 and QC1110 [16] and the β-galactosidase activity was measured by a described procedure [17].

### Epitope tagging of the *sod*C genes and immunodetection

Addition of 3xFLAG epitope tails at the ends of *sod*C genes was carried out according to a described procedure [18]. Briefly, a fragment containing the 3xFLAG epitope and kanamycin or chloramphenicol resistance cassette was amplified using oligonucleotides 3xFLAGcoli-F and 3xFLAGcoli-R on plasmid pSUB11 [18] or pSUB12 as template, respectively. pSUB12 is a derivative of pSUB11, which was obtained by the substitution of the original kanamycin resistance cassette with a *Xba*I-*Xba*I fragment from pKD3, carrying the chloramphenicol resistance cassette. These DNA fragments were independently electroporated into *E. coli *ED597 strain carrying pKD46; transformants were selected on LB plates containing the appropriate antibiotics. Recombination events were confirmed by PCR using the oligonucleotides 120ATGcoli-F and Kan-R or Clo-int (Table 2). The resulting strains were designated RG-F106 (*sodC*::3xFLAG-*kan*) and RG-F107 (*sodC*::3xFLAG-*cat*).

The same procedure was employed to construct RG-F108 (*sodC*-F1::3xFLAG-*kan*) and RG-F109 (*sodC*-F2::3xFLAG-*kan*) strains. In these cases, an identical PCR fragment, obtained with oligonucleotides 3xFLAG-F and 3xFLAG-R2 on pSUB11, was used to generate both the tagged strains. The same DNA fragment was also inserted into the RG-F106 (previously deleted of the kanamycin cassette) or RG-F107 strains, to obtain the doubly tagged strains RG-F110 (*sodC*::3xFLAG/*sodC*-F1::3xFLAG-*kan*) and RG-F111 (*sodC*::3xFLAG-*cat*/*sodC*-F2::3xFLAG-*kan*). Recombinations were confirmed by PCR using primers 240ATG-F1 or 240ATG-F2 and Kan-R on strains RG-F108, RG-F109, RG-F110 and RG-F111, while the couple of primers 120ATGcoli-F and Clo-int was used on RG-F110 and RG-F111.

s*odC *genes expression was analyzed using the epitope-tagged strains as a function of growth phase, at different temperatures of growth (25°C or 37°C), in bacteria cultivated in presence or absence of oxygen. Preparation of bacterial lysates from western-blot and immunodetection with anti-FLAG monoclonal antibodies (Sigma) was carried out as described [18]. Briefly, bacteria were harvested by centrifugation; pellets were resuspended in H_2_O, immediately mixed with Laemmli lysis buffer [19] and boiled for 10 minutes. The resulting lysates were quickly centrifuged to remove cell debris and subjected to 15% SDS-PAGE. Proteins were blotted onto nitrocellulose membrane (Hybond C, Amersham) and probed with antibodies. The epitope-flagged proteins were immunodetected by the use of anti-FLAG M2 monoclonal antibodies (Sigma-Aldrich) as the primary antibody and anti-mouse HRP-conjugated IgG (Bio-Rad) as the secondary antibody. Detection was performed by enhanced chemiluminescence (ECL Advance, Amersham).

### Cell culture studies

Synthesis of epitope-tagged SodC and SodC-F proteins was analyzed both in adherent and intracellular bacteria recovered from Caco-2 cells. Caco-2 cells (from a human colonic carcinoma) were maintained in D-MEM containing glucose 1 g/l, supplemented with 4 mM L-glutamine, 1% non-essential amino acids (NEAA) and 10% foetal calf serum in a humidified atmosphere of 5% CO_2 _at 37°C.

Infections of Caco-2 cells were performed essentially as previously described [20,21]. Confluent monolayers of epithelial cells were infected with RG-F111 strain (approximately 5 × 10^8^CFU/ml) grown overnight a 37°C in LB broth. Monolayers were then incubated in a humidified atmosphere of 5% CO_2 _at 37°C for 5 h. The inoculum was removed and the monolayer was washed (3×) with HBSS to remove non-adherent bacteria. Finally, the monolayer was disrupted by incubation with a solution of 1% Triton-X100 in PBS to release bacteria. The number of adherent bacteria was determined by plating 10-fold serial dilutions onto LB agar plates. Released bacteria were prepared for immunoblotting analysis as described above. For invasion studies, bacteria were allowed to adhere as described for 2 h at which point, the monolayers were washed twice with 37°C HBSS, then overlaid with cell culture medium supplemented with 100 μg/ml gentamicin and incubated for additional 3 h. The monolayers were then disrupted to release intracellular bacteria and CFU/ml determined.

### Isolation of *E. coli *O157:H7 EDL933 *sodC*-F1 and *sodC*-F2 and construction of expression vectors

The sequences corresponding to *sodC*-F1 and/or *sodC*-F2 genes of *E. coli *O157:H7 EDL933 were isolated by PCR amplification, using the chromosomal DNA (obtained from ATCC 700927D) as a template and the oligonucleotides O157-1For (which encompasses the ATG starting site) and O157-1Rev (downstream the translation stop codon) (Table 2). The amplified DNA fragments (about 560 bp) were digested with *Eco*RI and *Hind*III and inserted in the corresponding sites of pEMBL18 [22] to obtain pO157-F1 and pO157-F2, where the *sodC*-F1 and *sodC*-F2 coding sequences are under control of the *lacZ *promoter.

To analyze the properties of the signal peptide of prophagic *E. coli *O157:H7 SodC-F, the nucleotide sequence encoding the mature portion (lacking the signal peptide) of *sodC-*F1 was amplified with the oligonucleotides O157-2For and O157-2Rev, using pO157-F1 as a template. The amplified DNA fragment (about 470 bp) was digested with *Nco*I and *Eco*RI and cloned in pHEN-1 [23], obtaining the plasmid pO157-ΔCys encoding the mature prophagic enzyme lacking the leader sequence. In this vector the sequence encoding prophagic Cu,ZnSOD is fused to the leader peptide from the *pelB *gene of *Erwinia carotovora*.

### Extraction of lipid associated proteins

Soluble and membrane-associated proteins from *E. coli *71/18 [24] harbouring pEMBL18, pPLpEMBL18 [25], pO157-F1 or pO157-ΔCys were extracted as previously described [26,27].

Briefly, 200 μl of overnight cultures were inoculated into 10 ml of Luria-Bertani medium containing ampicillin (100 μg/ml), and the cultures were grown to a OD_600 _of 0.5 at 37°C. Then, IPTG, CuSO_4 _and ZnSO_4 _were added to a final concentration of 0.1 mM, 0.25 mM and 25 μM, respectively, and the cells were incubated for additional 3 hours at 37°C. Subsequently, bacterial cells (1.2 × 10^10^) were harvested by centrifugation (10000 × g, 5 min, at 4°C), washed with 1 ml of ice cold 10 mM sodium phosphate buffer, pH 7.0, suspended in 0.8 ml of the same buffer and sonicated. Cell debris was removed by centrifugation at 25000 × g for 10 min (repeated twice), and the supernatant, including membranes and cytosolic proteins, was subjected to Triton-X114 partitioning [26,27]. Triton-X114 and NaCl were added at a final concentration of 2% and 150 mM, respectively. The suspension was incubated overnight at 4°C on a rotating platform. Then, insoluble material was removed by centrifugation at 13000 × g for 10 min at 4°C. Subsequently, the temperature was raised to 37°C and the detergent and aqueous phases were separated by centrifugation at 13000 × g for 10 min at room temperature. The upper aqueous and lower detergent phases were both re-extracted once. Proteins from the detergent phase were recovered by precipitation with acetone (10 vol) at -20°C and resuspended in 50 mM Tris-HCl, pH 8.0, 150 mM NaCl and 1% Triton-X100.

The same procedure was used also to separate soluble and membrane-associated epitope-tagged proteins from *E. coli *O157:H7 strains. Proteins were subsequently separated by SDS-PAGE and analyzed by western blot.

### Protein expression and purification

Recombinant SodC was purified as described previously [28]. Soluble recombinant prophagic SodC-F1 was expressed and purified from the periplasm of *E. coli *QC871 [29] bearing the plasmid pO157-ΔCys. Bacteria were grown at 37°C in Luria-Bertani (LB) broth supplemented with ampicillin (100 μg/ml). When bacterial cells reached the mid-log phase, SodC-F1 expression was induced by the addition of 0.1 mM IPTG, 0.25 mM CuSO_4 _and 25 μM ZnSO_4_. After 16 h of growth, cells were harvested by centrifugation and the periplasmic proteins were extracted as described previously [30]. Proteins were extensively dialyzed against 20 mM Tris-HCl, pH 7.4, injected into a Hi-Load™16/10 Q-Sepharose fast protein liquid chromatography column (Amersham Biosciences), and fractionated with a 0–0.2 NaCl linear gradient. Subsequently, fractions containing the prophagic SodC-F1 protein were concentrated and dialyzed against 20 mM potassium phosphate buffer, pH 7.0 and subjected to cationic exchange chromatography on a Hi-Load™ 16/10 SP Sepharose (Amersham Biosciences) using a 0–0.2 NaCl linear gradient. Fractions containing the SodC-F were concentrated and dialyzed against 100 mM sodium phosphate buffer, pH 7.0, containing 1.5 M ammonium sulphate and loaded onto a Hi-Load™ Phenyl Sepharose FPLC column (Amersham Biosciences). Finally, the sample was concentrated to a small volume, dialyzed against 150 mM NaCl, 20 mM Tris-HCl, pH 7.4, and then injected into a Hi-Load™ 16/60 FPLC Superdex 75 gel filtration column (Amersham Biosciences) and eluted with the same buffer. At this stage, the protein appeared to be > 98% homogeneous, as judged by SDS-PAGE analysis. Protein concentration was evaluated by the method of Lowry [31] using bovine serum albumin as a standard. Paramagnetic copper content was determined by double integration of EPR spectra using a Cu(II)-EDTA solution as standard.

### Assays

Superoxide dismutase activity was assayed by the pyrogallol method [32]. One unit is defined as the amount of Cu,ZnSOD necessary to achieve 50% inhibition of pyrogallol autoxidation.

Proteinase K susceptibility of Cu,ZnSODs from *E. coli *O157:H7 ED597 was assayed incubating proteins at a concentration of 0.1 mg/ml at 37°C in 20 mM Tris-HCl, pH 6.8 or 8.0, in the presence of 0.1 mg/ml proteinase K. Aliquots were withdrawn at the indicated times and immediately assayed by the pyrogallol method to measure residual activity [32].

EDTA-dependent inactivation of Cu,ZnSODs was analyzed essentially as previously described [30]. Cu,ZnSOD samples at a concentration of 0.04 mg/ml were incubated at 37°C in 20 mM Tris-HCl, 0.1 mM EDTA, pH 6.8 or 8.0. Aliquots were withdrawn at different times and immediately assayed for residual activity by pyrogallol method [32].

To analyze susceptibility of deletion mutants to hydrogen peroxide, overnight bacterial cultures grown on LB medium were washed in PBS, diluted to 10^6 ^cells/ml and incubated in presence or absence of 250 μM hydrogen peroxide at 37°C. Aliquots were removed after 1 h and the number of viable cells was determined by serial dilution and plating onto LB agar. The percent survival following hydrogen peroxide exposure was calculated for each strain by the ratio of the CFU obtained upon incubation in PBS and the CFU obtained upon incubation in hydrogen peroxide. Each assays was repeated at least sixteen times, and standard deviations and Student's test were calculated.

### Differential scanning calorimetry

Heat capacity versus temperature profiles were obtained with a VP-DSC differential scanning calorimeter (MicroCal. Inc., Northampton, MA). Protein samples were dissolved at 0,2–0,4 mg/ml concentration, dialyzed against 0.1 M potassium phosphate buffer at the appropriate pH and degassed before the calorimetric experiment. The reference cell was filled with degassed dialysis buffer. Both cells were kept under an excess pressure of ~30 psi (~200 kPa) to avoid bubbling during the scan. A scan rate of 60°C/h was used in all the experiments. At the end of each run the solutions were cooled and subjected to a second heating cycle under the same conditions to determine the reversibility of the transitions. After each scan, samples were analyzed for integrity by SDS-PAGE and for residual activity using the pyrogallol method [32]. Thermograms were corrected by subtracting the instrumental base line, obtained with both cells filled with the same solvent, and normalized for protein concentration. Data analysis was performed with the ORIGIN software provided by MicroCal, after subtraction of a cubic base line connecting the pre- and post-transition traces.

## Results

The complete genome sequencing of the two *E. coli *O157:H7 epidemic strains EDL 933 [3] and Sakai [4] has revealed, in addition to the *sodC *gene typical of all *E. coli *strains, the presence of two *sod*C genes located in lambdoid prophage sequences. The two prophagic genes (s*odC*-F1 and s*odC*-F2) are identical except for a T/C mutation at nucleotide 335 downstream the ATG start codon, causing the replacement of a leucine residue (*sodC*-F1) with proline (*sodC*-F2). The mature proteins encoded by these two prophagic genes show reduced homology (61% identity), but similar size to the protein encoded by the chromosomal gene (155 and 154 amino acids, respectively). Also the regions that surround the two coding sequences are very similar and only a few mismatches are presents. Some of these are located around the nucleotides 240 and 550 upstream the start codon, and around nucleotide 500 downstream the stop codon. These regions have been chosen to design primers able to discriminate between the s*odC*-F1 and s*od*C-F2 genes in *E. coli *ED597 strain.

We have found that the nucleotide sequence of the DNA fragments amplified with the primers 550F1b and 500STOP1R and with the primers 550F2b and 500STOP2R (Table 2) on the genomic DNA from *E. coli *ED597 show a perfect identity with the *sodC*-F1 and *sodC*-F2 sequences of the EDL 933 strain, thus indicating a strict conservation of the *sodC *genes in these two bacterial strains.

### *sodC *expression studies

Expression of *E. coli *O157:H7 *sodC *genes was indirectly analyzed by monitoring protein accumulation in strains, which were modified by introducing the sequence encoding for the 3xFLAG epitope at the 3'end of each *sodC *gene within the chromosome. No differences in the accumulation of proteins were observed between strains RG-F108 and RG-F109, indicating that the two prophagic *sodC *genes, which share identical promoter sequences, are coregulated (data not shown). In contrast, differences in the intracellular amount of SodC and SodC-F proteins were observed under different conditions. To better appreciate the relative differences in expression between *sodC *and *sodC*-F, we constructed strains which bear the epitope-tagged copy of *sodC *and *sodC*-F1 (RG-F110 strain) or of *sodC *and *sodC*-F2 (RG-F111 strain). The relative abundance of epitope-flagged SodC and SodC-F proteins was measured in bacteria cultivated in LB broth under different conditions and in cultured epithelial cells.

**Figure 1:**
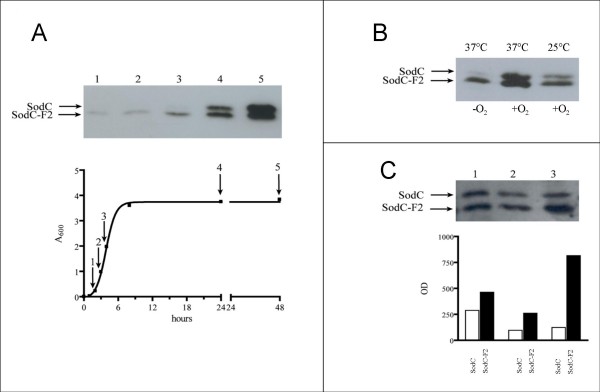
**SodC and SodC-F differential accumulation**. **Panel A: **Kinetics of accumulation of SodC and SodC-F2 proteins *in vitro *cultures. Overnight bacterial culture of strain RG-F111 was diluted 1:500 in LB medium (time zero) and grown at 37°C under aerobic conditions. Aliquots were withdrawn at the different times: 2 h (lane 1), 3 h (lane 2), 4 h (lane 3), 24 h (lane 4), and 48 h (lane 5). Cells were harvested, lysed and the samples (approximately 5 × 10^7 ^CFU lane^-1^) were loaded in a 15% SDS-PAGE and processed for the detection of epitope-tagged proteins as described in methods. **Panel B: **Effects of oxygen and temperature on SodC and SodC-F2 intracellular accumulation. Strain RG-F111 was grown for 24 h at 37°C (lanes 1 and 2) or at 25°C (lane 3) in presence (lanes 2 and 3) or absence of oxygen (lane 1) The samples were prepared as described above. **Panel C: **Variations in SodC and SodC-F2 relative abundance in infected Caco-2 cells. Strain RG-F111 was grown overnight in LB medium (lane 1) and adherent (lane 2) or intracellular (lane 3) bacteria were harvested from Caco-2 cells 5h post-infection. Approximately equal numbers of bacteria were loaded in each lane. A densitometric analysis of the western blot carried out with the Gel-Pro Analyzer software (Media Cybernetics) is reported in the graph. White bar, SodC; black bar, SodC-F2.

As shown in Figure 1 (panel A) both the enzymes are maximally expressed in the stationary phase, with SodC accumulation being hardly detectable in bacteria in the exponential phase of growth. In contrast, SodC accumulates at levels higher than those of SodC-F2 in bacteria recovered from starved cultures incubated for 48 hours at 37°C. Similar results were observed in RG-F106, RG-F107 and RG-110 confirming that there are not differences in the accumulation pattern of SodC-F1 and SodC-F2 (data not shown).

Abundance of SodC and SodC-F1/SodC-F2 was analyzed also in anaerobic cultures and as a function of the temperature of growth (25°C or 37°C) (Figure 1, panel B). The accumulation of SodC and SodC-F1/SodC-F2 is somehow altered either in bacteria cultivated at 37°C under anaerobic conditions (lane 1) or at 25°C in presence of oxygen (lane 3), but, apparently, the expression of SodC is much more affected by such conditions compared to SodC-F1 and SodC-F2.

As previous studies have revealed that *sodC *is regulated by the alternative sigma factor *rpoS *either in *E. coli *K12 [7] or in *S. *Typhimurium [10], we have studied gene expression with plasmids bearing fusions between the *sodC *and *sodC-*F1/*sodC*-F2 promoters and the β-galactosidase coding sequence, introducing these plasmids into wild type (QC771) and *rpoS *mutant (QC1110) *E. coli *strains. We have observed that the expression of *sodC *is drastically reduced in the *rpoS *mutant strain, whereas *sodC*-F is not regulated by this alternative sigma factor (data not shown).

To examine the levels of SodC and SodC-F proteins in adherent and intracellular bacteria, Caco-2 cells were infected with strain RG-F111. Panel C of Figure 1 compares the relative abundance of SodC and SodC-F2 in the bacterial lysates of stationary phase culture used to infect the cultured cells (lane 1), in adherent bacteria (lane 2) or intracellular bacteria harvested from epithelial cells 5 h post-infection (lane 3). A densitometric analysis of SodC-F2 and SodC abundance indicates that the relative ratio between the two proteins is close to 1.6 in bacteria grown in LB medium. SodC-F2 intracellular concentration is slightly enhanced in adherent bacteria (2.72), but significantly increases in intracellular bacteria, where, 5 hours post-infection, it is 6.57 folds more abundant than SodC.

### Susceptibility of *sod*C-mutants to hydrogen peroxide

**Figure 2:**
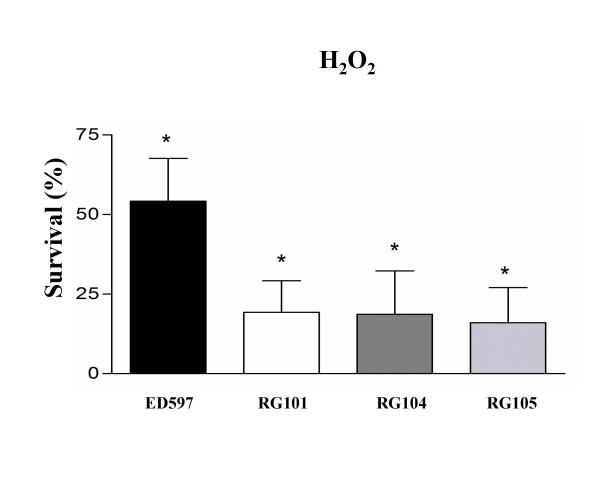
**Susceptibility of wild type and *sodC*-mutants strains to hydrogen peroxide**. Results are expressed as percent survival after 1 h incubation at 37°C. The percent survival was calculated for each strain by dividing the number of CFU/ml obtained from incubation in PBS alone by the number of CFU/ml obtained from incubation in 250 μM hydrogen peroxide. Values are means ± SD of at least sixteen independent experiments. A Student's *t *test analysis showed that the differences in survival between the wild type and the mutant strains were highly significant (*p < 0.01).

To investigate the role of Cu,ZnSOD in protection from exogenous oxidative stress, the effect of hydrogen peroxide on the survival of wild type and mutant strains. Experiments were carried out with bacteria grown to the stationary phase, to ensure the efficient expression of all *sodC *genes. Figure 2 shows that, upon a challenge with 250 μM hydrogen peroxide, all the mutant strains were killed at significantly higher rates with respect to the wild type strain. No significant differences were observed between the RG101, RG104 and RG105 strains, indicating the absence of an additive effect of *sodC *and *sodC*-F1/*sodC*-F2 deletion.

### Lipid modification signal in SodC-F1/SodC-F2

**Figure 3:**
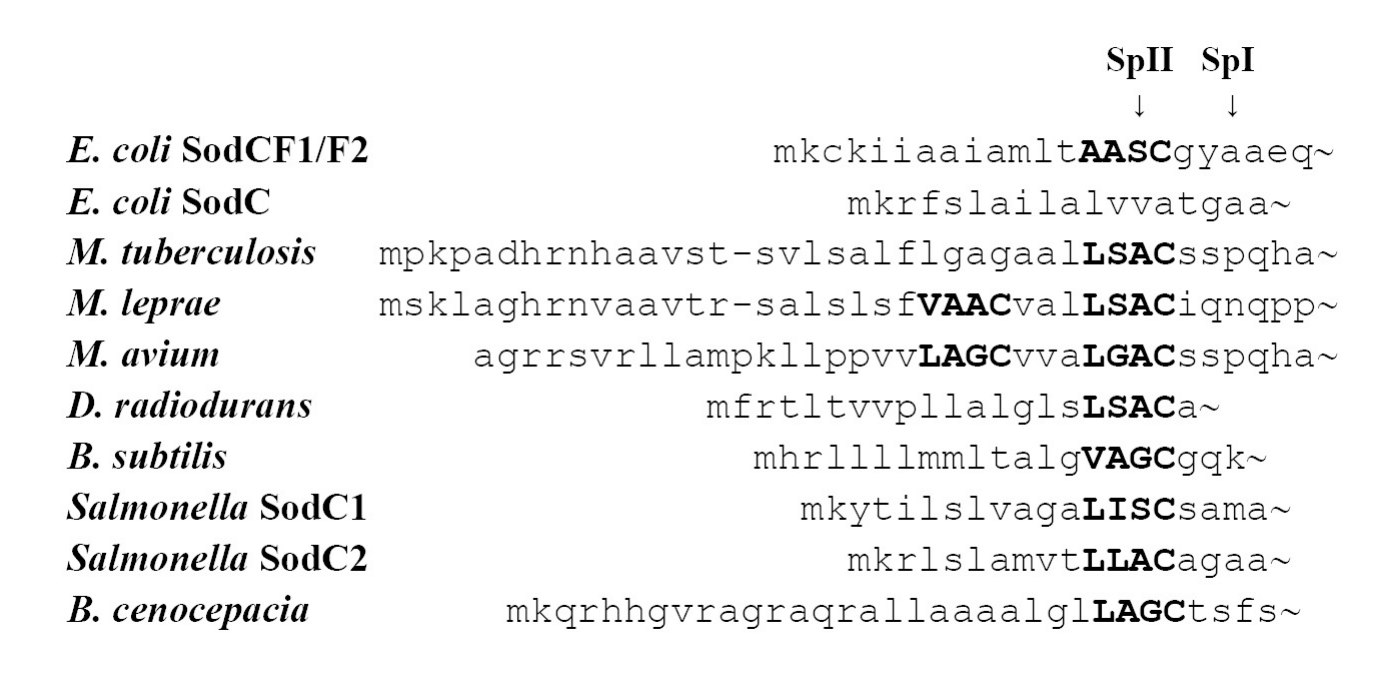
**Signal peptide sequences from bacterial Cu, ZnSODs**. Signal peptidase I and II cleavage sites were identified through the Lipo P program. The alignment of the signal peptides of bacterial Cu, ZnSOD is based on the Leu-X-Y-Cys motif.

The extracytoplasmic localization of bacterial Cu,ZnSODs is directed by typical signal peptide sequences recognized by specific membrane-associated peptidases. Signal peptidase I (SpI) removes the leader sequence from proteins which are to be exported in the periplasmic space or secreted in the extracellular milieu, while signal peptidase II (SpII) removes the leader peptide from lipid-modified precursor of exported proteins, which are targeted to the periplasmic inner and outer membranes. The N-terminal region of most SodC proteins from Gram-negative bacteria shows the typical features of the signal peptides recognized by SpI [33], whereas the signal sequences of mycobacterial SodC proteins are recognized by SpII [27]. Analysis of the amino acids sequences of SodC-F1 and SodC-F2 through the Lipo P program  showed that the leader peptide sequence of the two prophage encoded s*odC *genes possess features compatible with processing either by SpI or by SpII (Figure 3). SpI could cut the peptide between residues 20 and 21, whereas SpII could recognize the motif (lipobox) AASC and remove the first 16 residues, leaving a lipid-modified cysteine as N-terminal residue.

**Figure 4:**
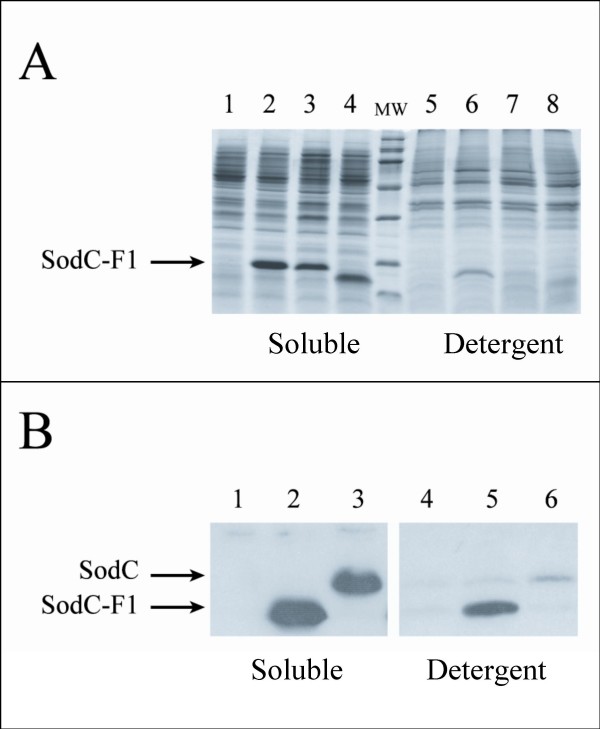
**Analysis of soluble and membrane-associated proteins**. **Panel A: **SDS-PAGE analysis of soluble (lanes 1–4) and membrane-associated proteins (lanes 5–8) from *E. coli *71/18 bearing pEMBL18 (lanes 1 and 5), pO157-F1 (lanes 2 and 6), pO157-ΔCys (lanes 3 and 7), and pPLpEMBL18 (lanes 4 and 8). **Panel B: **Soluble (lanes 1–3) and membrane-associated epitope-tagged proteins (lanes 4–6) from *E. coli *ED597 (lanes 1 and 4), RG-F108 (lanes 2 and 5) and RG-F106 (lanes 3 and 6) strains were extracted from cells grown at 37°C for 24 h. Each lane was loaded with material from approximately 5 × 10^7^CFU.

To verify if SodC-F1/SodC-F2 of *E. coli *O157:H7 can be lipid modified and, consequently, anchored to the bacterial membrane, we have initially separated soluble and membrane-associated recombinant proteins from *E. coli *71/18 cells [24] harbouring plasmids pO157-F1 or pO157-ΔCys by SDS-PAGE. pO157-ΔCys carries the sequence encoding mature prophagic SodC-F1 fused to a typical leader peptide recognized only by SpI. Figure 4 panel A shows that, while all the SodC-F1 protein expressed from cells bearing pO157-ΔCys is released in a soluble form in the periplasmic space (lanes 3 and 7), the protein expressed from the strain containing plasmid pO157-F1 is present both in the soluble (lane 2) and in the detergent phases (lane 6).

This results were confirmed by analysis of soluble and lipid-associated epitope-tagged proteins extracted from cells grown for 24 h incubation at 37°C from wild type, RG-F106 and RG-F108 *E. coli *O157:H7 strains. In fact, as shown in figure 4 panel B, SodC is present almost exclusively in the soluble fraction (lane 3), while the SodC-F1 is partitioned between the periplasmic space and the bacterial membrane (lanes 2 and 5). Similar results were obtained with RG-F107, RG-F109, RG-F110 and RG-F111 strains (data not shown). Thus, prophagic SodC-F1/SodC-F2 is present in the periplasmic space of *E. coli *O157:H7 either as a soluble protein or as a lipid-modified membrane protein.

### Biochemical characterization of prophagic SodC-F1

SodC-F1 and SodC-F2 have nearly identical amino acids sequences. Therefore we have cloned, overexpressed and characterized only one of the two proteins, namely SodC-F1. The apparent molecular weight of SodC-F1 determined by gel filtration chromatography is close to 30.6 kDa, roughly corresponding to the molecular weight expected for a dimeric enzyme (data not shown). As the only amino acidic difference between SodC-F1 and SodC-F2 does not involve a residue located at the dimer interface, it is likely that heterodimers between the two proteins may form *in vivo*. In contrast, SodC is a monomeric enzyme [34].

Previous studies have established that bacterial monomeric Cu,ZnSODs have quite different functional features with respect to dimeric enzymes of the same class [9,35]. For example, the *S. enterica *SodCI and SodCII proteins show remarkable differences in specific activity, protease resistance, metal affinity and peroxidative activity, with dimeric bacteriophage-encoded SodCI exhibiting superior stability and activity to monomeric SodCII [9]. To further analyze this issue, we have performed a detailed comparison of the *in vitro *biochemical properties of the two Cu,ZnSODs from *E. coli *O157:H7.

**Figure 5:**
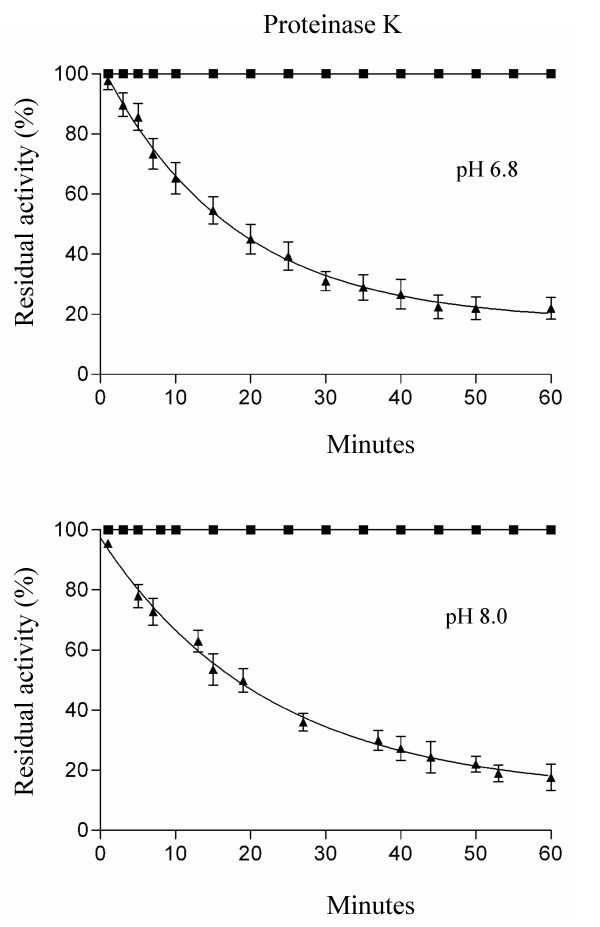
**Proteinase K susceptibility of SodC and SodC-F**. Proteins were incubated at 37°C in 20 mM Tris-HCl at pH 6.8 (**panel A**) or at pH 8.0 (**panel B**), in the presence of 0.1 mg/ml proteinase K. Aliquots were withdrawn at indicated times and immediately assayed for residual Cu, ZnSOD activity by the pyrogallol method: SodC (black square), SodC-F (black triangle). Each data point represents the mean of at least three independent measures.

While eukaryotic Cu,ZnSODs are highly resistant to proteolytic attack, all bacterial Cu,ZnSODs show susceptibility to proteolysis, monomeric variants showing higher sensitivity [9,35,36]. In agreement with previous investigations, we have found that dimeric SodC-F1 is highly resistant to proteinase K digestion, while monomeric SodC is rapidly inactivated both at pH 6.8 and 8.0 (Figure 5).

**Figure 6:**
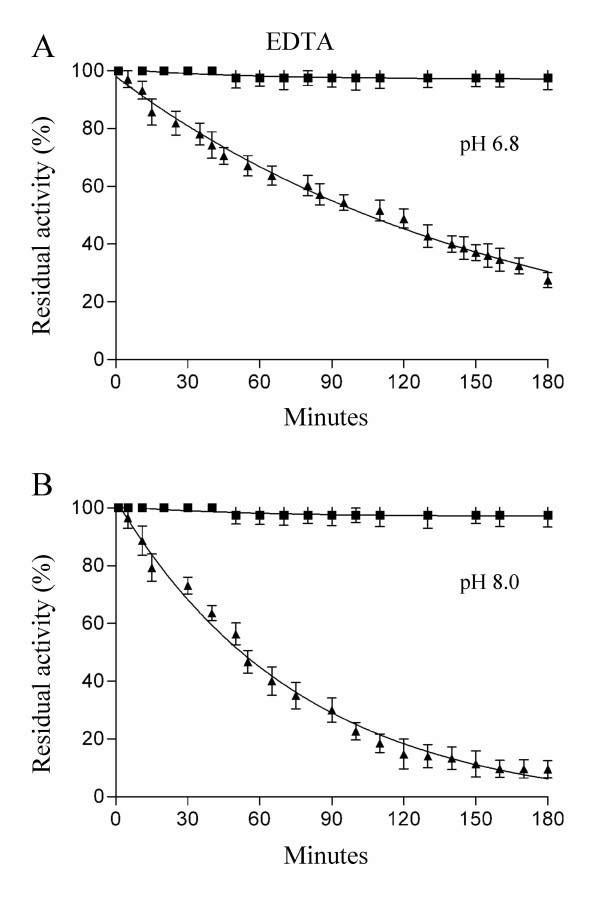
**EDTA-dependent inactivation of SodC and SodC-F**. Cu, ZnSOD samples at a concentration of 0.04 mg/ml were incubated at 37°C in 20 mM Tris-HCl buffer, 0.1 mM EDTA, pH 6.8 (**panel A**) or pH 8.0 (**panel B**). Aliquots were withdrawn at indicated times and assayed for residual Cu, ZnSOD activity by the pyrogallol method: SodC (black square), SodC-F (black triangle). Each data point represents the mean of at least three independent measures.

Unlike the eukaryotic enzymes which stably bind the active site metals, bacterial Cu,ZnSODs easily lose their cofactors and are therefore irreversibly inactivated in presence of EDTA [9,35,37,38]. The EDTA-mediated inactivation rates of bacterial enzymes depend on pH, being faster at alkaline pH [9,35,37] and on the quaternary structure stability [9,39]. Figure 6 shows the inactivation rates of SodC and SodC-F1 enzymes in 0.1 mM EDTA, 20 mM Tris-HCl, pH 6.8 (panel A) and pH 8.0 (panel B). Monomeric SodC from *E. coli *O157:H7 is rapidly inactivated, and the loss of activity is greater at pH 8.0 than at pH 6.8. On the contrary, SodC-F1 activity is not affected by incubation in the presence of EDTA, independently of pH.

As differences in the quaternary structures can influence Cu,ZnSOD activity, possibly through modulation of the active site conformational flexibility, we analyzed the catalytic activity of SodC and SodC-F1 by the pyrogallol method [32]. The monomeric SodC enzyme displays an activity of 6290 U/mg, while SodC-F1 shows an activity of 19380 U/mg.

**Figure 7:**
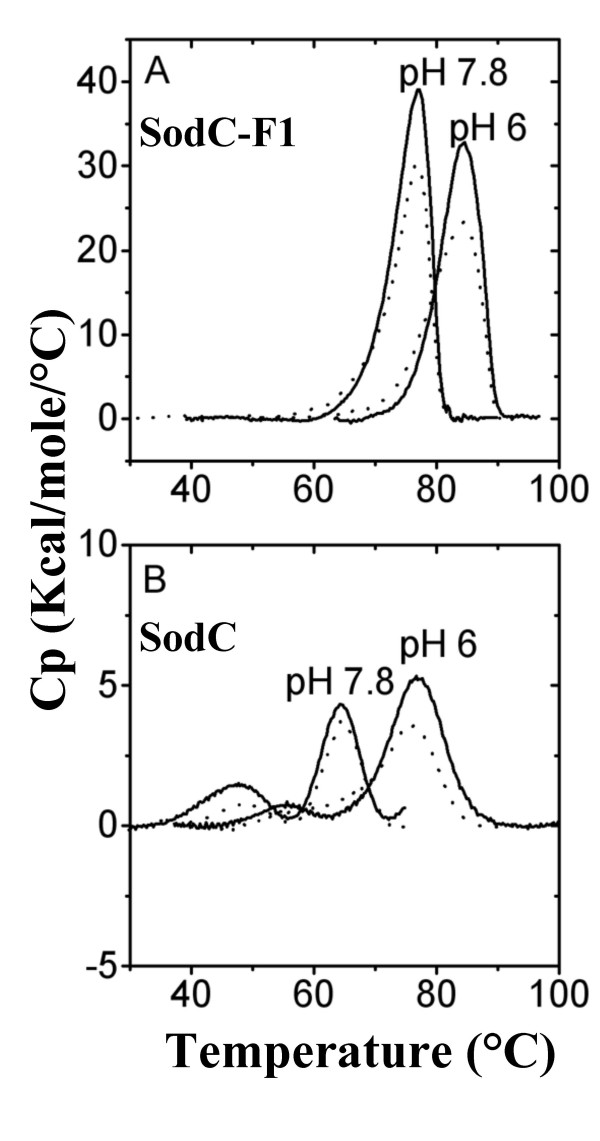
**Temperature dependence of the molar heat capacity of SodC and SodC-F**. Baseline-subtracted thermograms obtained at pH 6.0 and 7.8 for SodC-F (**panel A**) and SodC (**panel B**). Solid lines represent heating to 100°C, while dotted lines represent a second cycle of heating, after cooling to 10°C.

The thermal stability of the monomeric and dimeric *E. coli *O157:H7 Cu,ZnSODs was investigated by differential scanning calorimetry (DSC) under standardized experimental conditions (100 mM phosphate buffer, pH 6.0 and 7.8). The DSC profiles of the dimeric SodC-F1 protein are characterized by a single peak, which is centered around 87°C at pH 6.0 and around 78°C at pH 7.8 (Figure 7, panel A). As demonstrated by second scans of the same samples (dotted lines), the denaturation of the enzyme is highly reversible. In contrast, the thermogram of monomeric SodC is the sum of two independent two-state transitions, which have been previously shown to correspond to the apo and holo forms of the enzyme [37]. The transition corresponding to the most stable holo-enzyme, is centered around 77°C at pH 6.0 and around 64°C at pH. 7.8 (Figure 7, panel B).

## Discussion

Besides *E. coli *O157:H7, the presence of more than one *sodC *gene has been already described in several, although not all, *S. enterica *strains [10,40]. Genomic sequencing projects have revealed the presence of multiple *sodC *genes also in *Mycobacteriu*m *avium *104 (NC_008595), in the radioresistant bacterium *Deinococcus radiodurans *[41] and in the ancient microaerofilic bacterium *Aquifex aeolicus *[42]. Moreover, by an analysis of still incomplete genomes  we have observed that other *E. coli *strains, including the atypical enteropathogenic *E. coli *strains E110019 (serotype O111:H9) and B171 (serotype O111:NM) and the metal and antibiotic resistant environmental *E. coli *strain SECEC SMS-3-5 possess additional *sodC *copies showing high similarity with the *E. coli *O157:H7 *sodC*-F. In all the above mentioned microorganisms, Cu,ZnSODs could play a role in protecting cells from environmental reactive oxygen species, but this is not sufficient to explain the apparent redundancy of *sodC*. In order to better clarify this issue, we have undertaken a functional/structural characterization of the *sodC *genes of *E. coli *O157:H7 and their encoded proteins.

Inactivation of either the chromosomal *sodC *gene or *sodC*-F1 and *sodC*-F2 genes strongly decreases bacterial resistance to a challenge with hydrogen peroxide. This result, which is in line with previous studies carried out with different microorganisms [43-45], indicates that Cu,ZnSODs contribute to *E. coli *O157:H7 resistance against exogenous reactive oxygen species. However, the mutant strain lacking all the three *sodC *genes is not more susceptible to hydrogen peroxide than the strains lacking *sodC *or *sodC*-F1/*sodC*-F2. A similar lack of additivity of *sodC *mutations was previously observed in experiments carried out with *S*. Cholaeresuis *sodC *mutants [46]. It was suggested that a threshold level of superoxide dismutase activity is required to confer efficient protection against superoxide-mediated toxicity [46]. In this connection, it should be reminded that several studies have demonstrated that *sodC *mutants are not killed by exogenous superoxide but, although Cu,ZnSOD has no direct role in hydrogen peroxide scavenging, they are much more sensitive than wild type strains to treatments with hydrogen peroxide [43,44,47]. To explain the role of Cu,ZnSOD in hydrogen peroxide resistance it has been suggested that this oxidizing agent can react with superoxide generated within the periplasmic space to form the highly toxic hydroxyl radical via the Haber-Weiss reaction (H2O2 + O2^- ^→ O2 + OH^- ^+ OH.) [43]. Although this reaction proceeds slowly in the absence of a catalyst it can be stimulated by redox active metals which might be released in the periplasm due to hydrogen peroxide-mediated damage of metal-containing proteins [43]. It is possible that, under the very aggressive conditions represented by incubation of bacteria with high concentrations of hydrogen peroxide, the presence of all the Cu,ZnSODs is necessary to protect cellular targets which can be readily inactivated even by small amounts of hydroxyl radical formed in the periplasm of *sodC *mutants.

To evaluate the hypothesis that the presence of multiple Cu,ZnSODs in *E. coli *O157:H7 reflects differences in the regulation and/or in properties of the enzymes encoded by chromosomal and prophagic *sodC *genes, we have analyzed the intracellular accumulation of the epitope-tagged SodC and SodC-F proteins. We have observed that the chromosomally encoded SodC protein is mainly expressed during the stationary phase, under control of RpoS, and that its intracellular concentration is drastically reduced under anaerobic conditions or in bacteria cultivated at 25°C. This pattern of expression is very similar to that of SodC in non-pathogenic *E. coli *strains or of SodCII in *Salmonella *[9,10,44]. In contrast, the SodC-F proteins, while accumulating at maximal levels in starved cells, are detectable also in bacteria in the logarithmic phase and are not regulated by RpoS. Unlike SodC, SodC-F expression is not largely affected by oxygen. On the whole, the protein expression pattern of SodC-F is very similar to that previously described for the prophage-encoded *Salmonella *SodCI protein [9]. The match with the pattern of expression of the *sodC *genes of *Salmonella *was further completed by the observation that in intracellular bacteria recovered from Caco-2 cells the relative ratio between SodC and SodC-F was changed in favor of SodC-F. This could be due to differences in gene transcription and/or to a differential stability of the two enzymes in intracellular environments. To explore this possibility we have carried out a characterization of some functional/structural properties of the two proteins.

A first difference we have identified between SodC and SodC-F concerns their exact intracellular localization. As the Cu,ZnSODs from other Gram-negative bacteria, SodC is a fully soluble periplasmic protein. In contrast, a fraction of SodC-F is found in association to the bacterial membrane, likely due to processing of the signal peptide sequence by signal peptidase II. A similar lipid-modification of a superoxide dismutase has been previously reported for the enzyme from *M. tuberculosis *[27] which, however, lacks a true periplasmic space. Although, putative signal peptidase II cleavage sites have been previously recognized also in the Cu,ZnSODs from *S. enterica *[27] and *Burkholderia cenocepacia *[48], this is the first identification of a membrane-associated Cu,ZnSOD in a Gram-negative bacterium. It should be underlined that, the signal peptide of SodC-F is only partially processed by signal peptidase II, whereas a significant fraction of the enzyme is released in the periplasm following the removal of the transit peptide by signal peptidase I. It is tempting to speculate that such a partitioning of SodC-F between the periplasm and the membranes is useful to ensure a better protection of bacteria from exogenous radicals.

We have also compared some properties of purified SodC and SodC-F1. SodC-F1 shows higher catalytic activity than SodC. Moreover, dimeric SodC-F1 displays much higher resistance than monomeric SodC to inactivation mediated by proteolytic enzymes or by chelating agents. These properties are correlated with the higher conformational stability of SodC-F1 revealed by differential scanning calorimetry. In fact, we have observed a difference between the melting temperatures of the two enzymes of 10°C and 14°C at pH 6.0 and 7.8, respectively. It is worth nothing that the melting temperature of SodC-F1 is significantly higher than that of the dimeric bacterial enzyme from *Photobacterium leiognathi *[49] and approaches the values observed in the eukaryotic enzymes of this class [37], indicating that this is a very stable Cu,ZnSOD.

Taken together our data highlights a striking parallelism between the properties of *E. coli *O157:H7 SodC-F and those of *Salmonella *SodCI. In fact, both these genes have been acquired by virulent bacterial strains through bacteriophage-mediated processes, they show differences in their pattern of expression when compared to the chromosomal *sodC *gene and encode for dimeric proteins which are more stable and active than the monomeric SodC-enzyme.

Although further studies are required to clarify the exact role of the Cu,ZnSODs in *E. coli *O157:H7, this investigation supports the suggestion [9] that the chromosomally encoded SodC protein, which is present in all *E. coli *genomes, has a function that is largely unrelated to pathogenesis and likely protects the cell from endogenous superoxide produced during the aerobic metabolism. In contrast, the *sodC *genes carried by prophages, which can be identified only in a subset of bacterial strains, codify for proteins which have structural and regulatory features which likely favour bacterial survival within the hostile environments encountered during the infection.

## Conclusion

This work demonstrates that the *sodC *genes of *E. coli *O157:H7 are differently regulated and encode for proteins with distinct structural and functional properties, thus indicating that their functions are not truly redundant. These findings contribute to establish that the acquisition of additional *sodC *copies within bacterial genomes helps bacteria to expand their ability to withstand the reactive oxygen species generated within the host or in other hostile environments.

## Authors' contributions

MDO carried out protein characterization and expression studies in Caco-2 cells and participated in the design and writing of the manuscript; RS and RG carried out *E. coli *O157:H7 mutants construction; performed hydrogen peroxide resistance tests and *in vitro *expression studies, RG also participated in the design and writing of the manuscript; LN and GR participated in the design of the study and in the draft of the manuscript; LC carried out the DSC experiments; AB coordinated the study, participated in its design and in the writing of the manuscript. All authors read and approved the final manuscript.

## References

[B1] Dahan S, Knutton S, Shaw RK, Crepin VF, Dougan G, Frankel G (2004). Transcriptone of enterohemorrhagic *Escherichia coli *O157 adhering to eukaryotic plasma membranes. Infect Immun.

[B2] Kaper JB, Nataro JP, Mobley HL (2004). Pathogenic *Escherichia coli*. Nat Rev Microbiol.

[B3] Perna NT, Plunkett G, Burland V, Mau B, Glasner JD, Rose DJ, Mayhew GF, Evans PS, Gregor J, Kirkpatrick HA, Posfai G, Hackett J, Klink S, Boutin A, Shao Y, Miller L, Grotbeck EJ, Davis NW, Lim A, Dimalanta ET, Potamousis KD, Apodaca J, Anantharaman TS, Lin J, Yen G, Schwartz DC, Welch RA, Blattner FR (2001). Genome sequence of enterohaemorrhagic *Escherichia coli *O157:H7. Nature.

[B4] Hayashi T, Makino K, Ohnishi M, Kurokawa K, Ishii K, Yokoyama K, Han CG, Ohtsubo E, Nakayama K, Murata T, Tanaka M, Tobe T, Iida T, Takami H, Honda T, Sasakawa C, Ogasawara N, Yasunaga T, Kuhara S, Shiba T, Hattori M, Shinagawa H (2001). Complete genome sequence of enterohemorrhagic *Escherichia coli *O157:H7 and genomic comparison with a laboratory strain K-12. DNA Res.

[B5] Fridovich I (1995). Superoxide radical and superoxide dismutases. Ann Rev Biochem.

[B6] Battistoni A (2003). Role of prokaryotic Cu, Zn superoxide dismutase in pathogenesis. Biochem Soc Trans.

[B7] Korshunov S, Imlay JA (2006). Detection and quantification of superoxide formed within the periplasm of *Escherichia coli*. J Bacteriol.

[B8] Lynch M, Kuramitsu H (2000). Expression and role of superoxide dismutases (SOD) in pathogenic bacteria. Microbes Infect.

[B9] Ammendola S, Pasquali P, Pacello F, Rotilio G, Castor M, Libby SJ, Figueroa-Bossi N, Bossi L, Fang FC, Battistoni A (2008). Regulatory and structural differences in the Cu, Zn-superoxide dismutases of *Salmonella enterica *and their significance for virulence. J Biol Chem.

[B10] Fang FC, De Groote MA, Foster JW, Bäumler AJ, Ochsner U, Testerman T, Bearson S, Giard JC, Xu Y, Campbell G, Laessig T (1999). Virulent *Salmonella typhimurium *has two periplasmic Cu, Zn-superoxide dismutases. Proc Natl Acad Sci USA.

[B11] Krishnakumar R, Craig M, Imlay JA, Slauch JM (2004). Differences in enzymatic properties allow SodCI but not SodCII to contribute to virulence in *Salmonella enterica *serovar Typhimurium strain 14028. J Bacteriol.

[B12] Krishnakumar R, Kim B, Mollo EA, Imlay JA, Slauch JM (2007). Structural properties of periplasmic SodCI that correlate with virulence in *Salmonella enterica *serovar Typhimurium. J Bacteriol.

[B13] Figueroa-Bossi N, Ammendola S, Bossi L (2006). Differences in gene expression levels and in enzymatic qualities account for the uneven contribution of superoxide dismutases SodCI and SodCII to pathogenicity in *Salmonella enterica*. Microbes Infect.

[B14] Datsenko KA, Wanner BL (2000). One-step inactivation of chromosomal genes in *Escherichia coli *K-12 using PCR products. Proc Natl Acad Sci USA.

[B15] Casadaban MJ, Chou J, Cohen SN (1980). In vitro gene fusions that join an enzymatically active beta-galactosidase segment to amino-terminal fragments of exogenous proteins: *Escherichia coli *plasmid vectors for the detection and cloning of translational initiation signals. J Bacteriol.

[B16] Battistoni A, Pacello F, Folcarelli S, Ajello M, Donnarumma G, Greco R, Ammendolia MG, Touati D, Rotilio G, Valenti P (2000). Increased expression of periplasmic Cu, Zn superoxide dismutase enhances the survival of *Escherichia coli *invasive strains within nonphagocytic cells. Infect Immun.

[B17] Sambrook J, Fritsch EF, Maniatis T (1989). Molecular Cloning A Laboratory Manual.

[B18] Uzzau S, Figueroa-Bossi N, Rubino S, Bossi L (2001). Epitope tagging of chromosomal genes in *Salmonella*. Proc Natl Acad Sci USA.

[B19] Laemmli UK (1970). Cleavage of structural proteins during the assembly of the head of bacteriophage T4. Nature.

[B20] Aktan I, Sprigings KA, La Ragione RM, Faulkner LM, Paiba GA, Woodward MJ (2004). Characterisation of attaching-effacing *Escherichia coli *isolates from animals at slaughter in England and Wales. Vet Microbiol.

[B21] La Ragione RM, Best A, Sprigings KA, Cooley WA, Jepson MA, Woodward MJ (2004). Interaction between attaching-effacing *Escherichia coli *serotypes O157:H7 and O26:K60 in cell culture. Vet Microbiol.

[B22] Dente L, Cesareni G, Cortese R (1983). pEMBL: a new family of single stranded plasmids. Nucleic Acids Res.

[B23] Hoogenboom HR, Griffiths AD, Johnson KS, Chiswell DJ, Hudson P, Winter G (1991). Multi-subunit proteins on the surface of filamentous phage: methodologies for displaying antibody (Fab) heavy and light chains. Nucleic Acids Res.

[B24] Messing J, Gronenborn B, Müller-Hill B, Hans Hopschneider P (1977). Filamentous coliphage M13 as a cloning vehicle: insertion of a HindII fragment of the lac regulatory region in M13 replicative form in vitro. Proc Natl Acad Sci USA.

[B25] Foti D, Lo Curto B, Cuzzocrea G, Stroppolo ME, Polizio F, Venanzi M, Desideri A (1997). Spectroscopic characterization of recombinant Cu, Zn superoxide dismutase from *Photobacterium leiognathi *expressed in *Escherichia coli*: evidence for a novel catalytic copper binding site. Biochemistry.

[B26] Bordier C (1981). Phase separation of integral membrane proteins in Triton X-114 solution. J Biol Chem.

[B27] D'Orazio M, Folcarelli S, Mariani F, Colizzi V, Rotilio G, Battistoni A (2001). Lipid modification of the Cu, Zn superoxide dismutase from *Mycobacterium tuberculosis*. Biochem J.

[B28] Battistoni A, Folcarelli S, Gabbianelli R, Capo C, Rotilio G (1996). The Cu, Zn superoxide dismutase from *Escherichia coli *retains monomeric structure at high protein concentration. Evidence for altered subunit interaction in all the bacteriocupreins. Biochem J.

[B29] Carlioz A, Touati D (1986). Isolation of superoxide dismutase mutants in *Escherichia coli*: is superoxide dismutase necessary for aerobic life?. EMBO J.

[B30] Battistoni A, Donnarumma G, Greco R, Valenti P, Rotilio G (1998). Overexpression of a hydrogen peroxide-resistant periplasmic Cu, Zn superoxide dismutase protects *Escherichia coli *from macrophage killing. Biochem Biophys Res Commun.

[B31] Lowry OH, Rosebrough NJ, Farr AL, Randall RJ (1951). Protein measurement with the Folin phenol reagent. J Biol Chem.

[B32] Marklund S, Marklund G (1974). Involvement of the superoxide anion radical in the autoxidation of pyrogallol and a convenient assay for superoxide dismutase. Eur J Biochem.

[B33] Gabbianelli R, Signoretti C, Marta I, Battistoni A, Nicolini L (2004). *Vibrio cholerae *periplasmic superoxide dismutase: isolation of the gene and overexpression of the protein. J Biotechnol.

[B34] Battistoni A, Rotilio G (1995). Isolation of an active and heat-stable monomeric form of Cu, Zn superoxide dismutase from the periplasmic space of *Escherichia coli*. FEBS Lett.

[B35] Gabbianelli R, D'Orazio M, Pacello F, O'Neill P, Nicolini L, Rotilio G, Battistoni A (2004). Distinctive functional features in prokaryotic and eukaryotic Cu, Zn superoxide dismutases. Biol Chem.

[B36] Falconi M, Parrilli L, Battistoni A, Desideri A (2002). Flexibility in monomeric Cu, Zn superoxide dismutase detected by limited proteolysis and molecular dynamics simulation. Proteins.

[B37] Battistoni A, Folcarelli S, Cervone L, Polizio F, Desideri A, Giartosio A, Rotilio G (1998). Role of the dimeric structure in Cu, Zn superoxide dismutase. pH-dependent, reversible denaturation of the monomeric enzyme from *Escherichia coli*. J Biol Chem.

[B38] Stroppolo ME, Pesce A, D'Orazio M, O'Neill P, Bordo D, Rosano C, Milani M, Battistoni A, Bolognesi M, Desideri A (2001). Single mutations at the subunit interface modulate copper reactivity in *Photobacterium leiognathi *Cu, Zn superoxide dismutase. J Mol Biol.

[B39] D'Orazio M, Battistoni A, Stroppolo ME, Desideri A (2000). Single mutation induces a metal-dependent subunit association in dimeric Cu, Zn superoxide dismutase. Biochem Biophys Res Commun.

[B40] Figueroa-Bossi N, Uzzau S, Maloriol D, Bossi L (2001). Variable assortment of prophages provides a transferable repertoire of pathogenic determinants in *Salmonella*. Mol Microbiol.

[B41] White O, Eisen JA, Heidelberg JF, Hickey EK, Peterson JD, Dodson RJ, Haft DH, Gwinn ML, Nelson WC, Richardson DL, Moffat KS, Qin H, Jiang L, Pamphile W, Crosby M, Shen M, Vamathevan JJ, Lam P, McDonald L, Utterback T, Zalewski C, Makarova KS, Aravind L, Daly MJ, Minton KW, Fleischmann RD, Ketchum KA, Nelson KE, Salzberg S, Smith HO, Venter JC, Fraser CM (1999). Genome sequence of the radioresistant bacterium *Deinococcus radiodurans *R1. Science.

[B42] Deckert G, Warren PV, Gaasterland T, Young WG, Lenox AL, Graham DE, Overbeek R, Snead MA, Keller M, Aujay M, Huber R, Feldman RA, Short JM, Olsen GJ, Swanson RV (1998). The complete genome of the hyperthermophilic bacterium *Aquifex aeolicus*. Nature.

[B43] Pacello F, Ceci P, Ammendola S, Pasquali P, Chiancone E, Battistoni A (2008). Periplasmic Cu, Zn superoxide dismutase and cytoplasmic Dps concur in protecting *Salmonella *enterica serovar Typhimurium from extracellular reactive oxygen species. Biochim Biophys Acta.

[B44] Gort AM, Ferber DM, Imlay JA (1999). The regulation and role of the periplasmic copper, zinc superoxide dismutase of *Escherichia coli*. Mol Microbiol.

[B45] Kim YH, Lee Y, Kim S, Yeom J, Yeom S, Seok Kim B, Oh S, Park S, Jeon CO, Park W (2006). The role of periplasmic antioxidant enzymes (superoxide dismutase and thiol peroxidase) of the Shiga toxin-producing *Escherichia coli *O157:H7 in the formation of biofilms. Proteomics.

[B46] Sansone A, Watson PR, Wallis TS, Langford PR, Kroll JS (2002). The role of two periplasmic copper- and zinc-cofactored superoxide dismutases in the virulence of *Salmonella choleraesuis*. Microbiology.

[B47] Steinman HM (1993). Function of periplasmic copper-zinc superoxide dismutase in *Caulobacter crescentus*. J Bacteriol.

[B48] Keith KE, Valvano MA (2007). Characterization of SodC, a periplasmic superoxide dismutase from *Burkholderia cenocepacia*. Infect Immun.

[B49] Bourne Y, Redford SM, Steinman HM, Lepock JR, Tainer JA, Getzoff ED (1996). Novel dimeric interface and electrostatic recognition in bacterial Cu, Zn superoxide dismutase. Proc Natl Acad Sci USA.

